# Identification and Biological Characteristics of *Alternaria gossypina* as a Promising Biocontrol Agent for the Control of *Mikania micrantha*

**DOI:** 10.3390/jof10100691

**Published:** 2024-10-03

**Authors:** Lichen Feng, Lianrong Hu, Jingyi Bo, Mei Ji, Sangzi Ze, Yan’e Ding, Bin Yang, Ning Zhao

**Affiliations:** 1 College of Biological Science and Food Engineering, Southwest Forestry University, Kunming 650224, China; flc225169@163.com (L.F.); 15223788948@163.com (J.B.); longnanding@163.com (Y.D.); 2 Yunnan Academy of Forestry and Grassland, Kunming 650224, China; hulianrong@yafg.ac.cn (L.H.); meiji.emma@163.com (M.J.); 3 Yunnan Forestry and Grassland Pest Control and Quarantine Bureau, Kunming 650051, China; zesangzi@163.com; 4 School of Biological and Chemical Science, Pu’er University, Pu’er 665000, China; 5 Key Laboratory of Forest Disaster Warning and Control of Yunnan Province, Southwest Forestry University, Kunming 650224, China

**Keywords:** *Mikania micrantha*, *Alternaria gossypina*, fungal herbicides, biological characteristics

## Abstract

*Mikania micrantha* is one of the most threatening invasive plant species in the world. Its invasion has greatly reduced the species diversity of the invaded areas. The development of fungal herbicides using phytopathogenic fungi has attracted considerable attention in recent years. In this study, a tissue isolation method was used to isolate and screen the strain SWFU-MM002 with strong pathogenicity to *M. micrantha* leaves from naturally occurring *M. micrantha*. Through morphological observation, *ITS*, *GAPDH*, and *Alta-1* gene sequence homology, we compare and construct a phylogenetic tree to determine their taxonomic status. In addition, the biological characteristics of strain SWFU-MM002 were studied. The results showed that, combined with morphological and molecular biology identification, the strain was identified as *Alternaria gossypina*; biological characteristic research showed that the optimal medium for the growth of mycelium of this strain is PDA medium. At the optimal temperature of 27 °C and pH between 6 and 10, the mycelium can grow well. The best carbon and nitrogen sources are maltose and peptone, respectively. Analysing the infection process under a light microscope showed that SWFU-MM002 mycelia invaded the leaf tissue through stomata and colonized, eventually causing damage to the host. This is the first report of leaf spot of *M. micrantha* caused by *A. gossypina*. This study can lay a solid foundation for the development of *A. gossypina* as a control agent for *M. micrantha*.

## 1. Introduction

*Mikania micrantha* Kunth (Asteraceae) is a perennial vine of the genus *Mikania* in the Compositae family [1]. It is one of the top 100 most threatening invasive species in the world [2,3] and is one of the agricultural invasive species that China is focusing on controlling [4]. *M. micrantha* is native to Central and South America and later spread to the Pacific region [5]. In the early 1920s, *M. micrantha* first appeared as a weed in Hong Kong, China, and has since spread to inland China [6]. It is now widely distributed in Guangdong, Hainan, Guangxi, Yunnan, Fujian, and other provinces, and there is a trend of further expansion. When *M. micrantha* invades, it covers the surface of other plants, secreting allelochemicals that reduce the light available to the plant and thus inhibit the growth of the plant or even kill the plant [7]. It not only reduces the local species diversity and destroys the species structure and function of the invaded area, but also changes the physical and chemical properties of the soil in the invaded area, which has a great impact on the local agriculture and forestry [8,9,10,11]. At present, *M. micrantha* prevention and control mainly adopts comprehensive control methods that combine quarantine monitoring, physical control, chemical control, biological control, and community modification [3]. However, physical control consumes a lot of manpower and material resources and is prone to relapse. Long-term use of chemical agents will cause environmental pollution and increase weed resistance. Therefore, compared with other control methods, biological control has the characteristics of environmental friendliness and high safety [12,13,14].

Microbial herbicides have attracted much attention in recent years because of their low toxicity, easy degradation, and abundant sources [15]. The main sources of microbial herbicides are plant pathogenic living microorganisms and microbial secondary metabolites and their derivatives. Plant pathogenic living microorganisms and their preparations are mainly fungi, actinobacteria, bacteria, and viruses. Microbial metabolites and their derivatives mainly include polypeptides, terpenes, macrolides, and phenols [16]. Plant pathogenic fungi are the main pathogenic microorganisms that cause diseases in plants, and once a disease occurs, it can cause a large reduction in crop yields and bring great economic losses to agricultural production [17]. However, for farmland weeds, plant pathogens are good resources of microbial herbicides, which do not pollute the environment and do not make weeds resistant [18,19]. On the one hand, plant pathogenic fungi generally infect only one kind of weed or its related species, and are relatively friendly to the surrounding ecological environment. On the other hand, plant pathogenic fungi will produce toxins, which will destroy the physiological structure of the host [20], which is conducive to pathogenic infection and enhances the biological control effect of weeds [21,22]. Therefore, the research and development of microbial herbicides have broad prospects, and there are mainly nine genera of fungi with herbicide research and development value: *Colleototrichum*, *Sclreotinia*, *Phytophthora*, *Entyloma*, *Alternaria*, *Fusarium*, *Puccinia*, *Ascochyta*, and *Cercospora* [23,24]. Since the 20th century, more and more scholars have begun to use plant fungi to develop herbicides and evaluate the herbicidal potential of biocontrol fungi.

There are many species of *Alternaria*; it belongs to Fungi, Ascomycota, Dothideomycetes, Pleosporales, Pleosporaceae [25]. It is highly adaptable, widely distributed around the world, and is one of the most common pathogens in ecosystems [26]. *Alternaria* can infect wheat [27], onion [28], and potato [29] in the field and cause crop yield decrease. Infecting ornamental plants, Chinese herbs and so on, causing serious economic losses [30]. More importantly, *Alternaria* is able to target damage to target plants, so using *Alternaria* to develop a new type of pesticide–bioherbicide, which is less toxic and long-lasting, is in line with current advocacy of sustainable development in agriculture. In the aspect of biological control of weeds, *Alternaria* also shows broad application prospects, and more than 10 species of *Alternaria* have been reported for weed control. Abdessemed et al. reported that inoculation with oil emulsion containing 10^6^ spores/mL had good preventive effect on *Xanthium strumarium*, and the preventive effect on *X. strumarium* reached more than 95% [31]. Saxena and Kumar applied 10^6^ spores/mL of *Alternaria alternata* ITCC4896 to achieve 50% efficacy against *Parthenium hysterophorus* after 96 h and 100% after 7 d [32]. In addition, the secondary metabolites produced by *Alternaria* are widely used in the control of weeds in farmland [21,33].

The study of fungal biological characteristics is an indispensable part of the study of fungal herbicides. It will help us to understand the culture conditions of pathogenic fungi and lay a foundation for the further development and application of fungal herbicides. In this study, natural disease of *M. micrantha* plants collected in Ruili, Yunnan province, were isolated by tissue separation, and a highly pathogenic strain, SWFU-MM002, was selected as the research object. Its classification status was determined by morphological and molecular biological analysis, and the optimal culture medium, carbon source, nitrogen source, and pH and light conditions for its growth were screened to explore the optimal culture conditions. This provides a theoretical basis for the development and research of *M. micrantha* microbial herbicides.

## 2. Materials and Methods

### 2.1. Isolation and Purification of Pathogens

In 2023, leaf spot disease was observed on *M. micrantha* leaves in Ruili, Yunnan Province, China. The junction tissues of diseased and healthy parts of the leaves were selected and the pathogenic fungi were isolated and purified by the tissue separation method. Briefly, the leaves with lesions were cleaned with sterile water, the moisture was absorbed, and [(3~5) mm × (3~5) mm] of the disease area was excised. The samples were then surface sterilized in 75% ethanol for 1 min, rinsed 3 times with sterile water, and dried with sterile filter paper. Subsequently, the diseased leaves were placed onto potato dextrose agar (PDA) medium and were incubated at 27 °C in the dark until mycelia appeared. The mycelia surrounding the disinfected leaves were selected for further purification; pure cultures were obtained after more than 3 successive subcultures and then stored at 4 °C for further study.

### 2.2. Pathogenicity Tests on Host Leaves

The isolated and purified pathogen strains were inoculated on the PDA and incubator at 27 °C for 7 days. After that, a sterile hole puncher was used to take mycelium pieces from the edge of the colony. The leaves of *M. micrantha* with the same growth status were inoculated with sterile needle puncture wounds, and then the mycelium block (d = 5 mm) was inoculated to the wound of *M. micrantha* leaves for back infection. The leaves were cultured at 27 °C to observe whether there were disease spots on the leaves. After the appearance of disease spots, the leaves were removed and the pathogens were separated and identified again to fulfil Koch’s postulates.

### 2.3. Disease Severity Assessments

We used a sterile punch to take out a mycelium block (d = 5 mm) from the edge of the colony that had been cultured for 5 days and placed the side with the mycelium in the middle of the leaf. Each leaf was inoculated with one mycelium block and leaves inoculated with sterile media blocks were used as controls in five biological replicates. The inoculated mycelial blocks were removed after 2 days, and the disease status of the leaves was observed 4 days later and the disease grading statistics were determined. The severity assessment was rated after inoculation by using a scale containing 6 grades (Table 1).

PDI = {Σ(N ×V)/(N × S)} × 100

where Σ is summation, N is the no. of leaves in each category, V is the numerical value of leaves observed, and S is the maximum numerical value/grade.

### 2.4. Morphological Identification of the Pathogen

The purified strain SWFU-MM002 was inoculated into the central position of PDA medium and cultured under darkness at 27 °C. The morphological characteristics of the colony were observed, photographed, and recorded every day. At the same time, microscopic observations were made using optical microscopy, such as colony characteristics, hyphal structures, and sporangiospores, which were preserved and recorded by photographing. The species status of the pathogen of this leaf spot disease was determined by analyzing colony characteristics and conidial morphological features.

### 2.5. Molecular Identification of Fungal Isolates

DNA extraction was performed with the Fungal Genomic DNA Rapid Extraction Kit, and PCR reactions were performed using the ribosomal RNA internal transcribed spacer region gene *ITS*, the streptozotocin allergen gene *Alta-1*, and the 3-phosphoglyceraldehyde dehydrogenase gene *gpd* (Table 2). The amplified products were sent to Sequencing by Tsingke Biotech Co., Ltd., Beijing, China. The sequences obtained from sequencing were submitted to NCBI to obtain the registration number and download the information such as close strains to the sequences of the three genes mentioned above, a comparison of the strains, and the registration numbers of the related genes (Table S1). Sequence comparison, Gblock trimming, and Concatenation stringing were performed by MAFFT in Phylosuite software. On this basis, a phylogenetic tree was constructed with the help of neighbour-joining (NJ) in MEGA7.0 software.

### 2.6. Biological Characterisation Studies

#### 2.6.1. Effect of Different Media on Mycelial Growth

The activated mycelial clumps were uniformly intercepted with a sterile perforator (5 mm in diameter) on an ultra-clean workbench, inoculated on 10 different types of media (PDA, PSA, LB, Czapek, PMA, Martin’s medium, SDAY, PSKA, Richard, and modified Fries’ medium), and placed in a thermostatic incubator at 27 °C under dark conditions. Five replicates of each medium were incubated and colony diameter was measured with a vernier calliper during incubation.

#### 2.6.2. Effect of Different pH Values on Mycelial Growth

The pH of the PDA medium was adjusted to 4, 5, 6, 7, 8, 9 and 10 using 0.1 mol/L HCl and 0.1 mol/L NaOH solutions for mixing, and the mycelium blocks were intercepted and inoculated into the centre of the Petri dish and placed in a constant-temperature incubator at 27 °C under dark conditions for incubation, observation, and recording. Colony diameter was measured after 7 days to assess the growth status of the strain.

#### 2.6.3. Effect of Different Temperature on Mycelial Growth

After inoculating the mycelial mass on PDA medium, the pathogen was placed in constant-temperature culture at 27 °C for 7 d. A sterile punch with a diameter of 5 mm was used to punch a number of patties at the edge of the colony, which were inoculated on PDA medium for culture, and the culture temperature was set at 5, 10, 15, 20, 25, 27, 30, and 35 °C for a total of 8 temperature gradients for constant dark culture, with 5 replications for each treatment, observation, and recording. Colony diameter was measured after 7 days to assess the growth status of the strain.

#### 2.6.4. Mycelial Growth Characteristics with Different Carbon and Nitrogen Sources

Czapek medium was used as the base medium, and equal amounts of glucose, fructose, maltose, lactose, soluble starch, glycerol, and mannitol were used as the sole carbon sources to replace sucrose in the medium formulation; equal amounts of beef paste, peptones, urea, ammonium nitrate, and aminoacetic acid were selected as the sole nitrogen sources to replace sodium nitrate in the medium formulation. The cuttings were placed in the centre of each carbon-source medium plate and incubated in the dark in a thermostatic incubator at 27 °C for observation and recording, and colony diameters were measured after 7 days to assess the growth status of the strains.

#### 2.6.5. Effect of Different Light Conditions on Mycelial Growth

After the pathogen was incubated at 27 °C for 7 d, a 5 mm diameter sterile punch was used to punch a number of cakes at the edge of the colony, which were inoculated on PDA medium and then placed in a 27 °C light incubator (16 h of light/8 h of darkness); one treatment was wrapped in tin foil to perform the dark culture, and the other one was not wrapped in tin foil, i.e., light and dark cultures were alternated, and five replications were set for each treatment. We observed and recorded the growth of mycelium. Colony diameter was measured after 7 days to assess the growth status of the strain.

### 2.7. Observation of Leaf Infection Process of M. micrantha by Strain SWFU-MM002

The mycelium of strain SWFU-MM002 was inoculated on the leaves of *M. micrantha*. The leaf materials were taken at 6 h, 12 h, 24 h, 48 h, 72 h and 96 h, and the leaves were cut into small pieces with scissors. The samples were immersed in FAA fixing solution (70% ethanol/formaldehyde/Acetic acid = 18:1:1) and were fixed for 24 h; then, they were removed and put into decolorizing solution (saturated chloral hydrate aqueous solution) to be made transparent for 24 h, until the leaves were completely decolorized, and then washed with water. The growth of mycelia and the formation of conidia were observed under an optical microscope after soaking and staining with lactic acid phenol blue solution.

### 2.8. Statistical Analysis

Experimental data were analyzed using IBM SPSS Statistics 27.0.1 software for variance analysis and difference analysis between different treatments, and Graph Pad Prism version 8.0 (Graph Pad Software, San Diego, CA, USA) was used for graphing.

## 3. Results

### 3.1. Natural Symptoms and Pathogen Isolation

**Figure 1:**
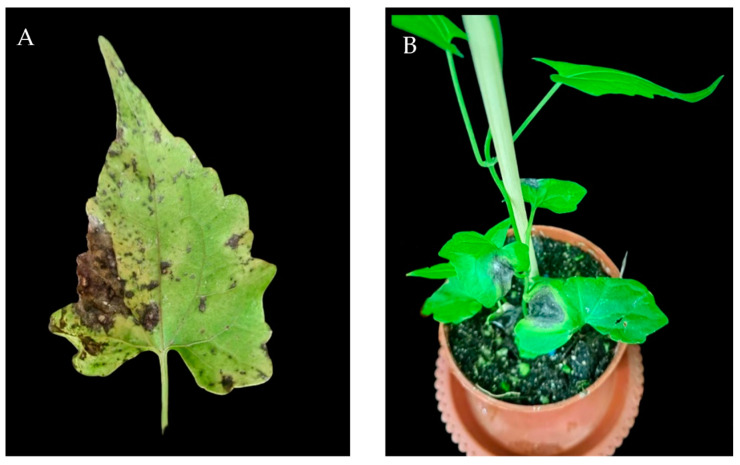
Symptoms of *Alternaria* development on leaves of *Mikania micrantha*. (**A**) Symptoms on diseased leaves in nature; (**B**) leaf symptoms after indoor inoculation.

Symptoms of *M. micrantha* leaf pathogenesis: First, small black spots formed on the leaf blades; then, the spots became larger and formed irregular features; the leaf blades around the spots turned yellow; the affected area increased in the later stage; the centre of the spots became greyish-white; and the leaf blades wilted (Figure 1A). The isolated pathogen was inoculated onto the leaves of live *M. micrantha* plants to determine pathogenicity, and mycelium was found growing from the puncture wounds of the pathogen-inoculated leaves after 2 d. After 7 d, the leaves showed black browning spots (Figure 1B), with clear boundaries with the healthy parts, and the leaves of the control group did not show the disease. To confirm Koch’s hypothesis, the pathogen was successfully re-isolated from infected leaves with morphological characteristics and genetic sequences matching those of the original isolates.

### 3.2. Pathogenicity Studies

After the strain SWFU-MM002 was inoculated on the back of *M. micrantha* leaves for 4 days, a large number of mycelia grew around the cake, and the mycelium penetrated the leaf cells and grew to the leaf surface. Slight yellow and green phenomena appeared on the leaves around the cake, and brown spots formed at the inoculation site. At this time, the disease index was 25.713, and no changes were observed in the control group. After 15 days of inoculation, the lesion area gradually spread outward, the leaves turned black and rotten, and the disease index reached 75.74, indicating that the strain SWFU-MM002 had strong pathogenicity against *M. micrantha*.

### 3.3. Morphology of Fungal Isolates

**Figure 2:**
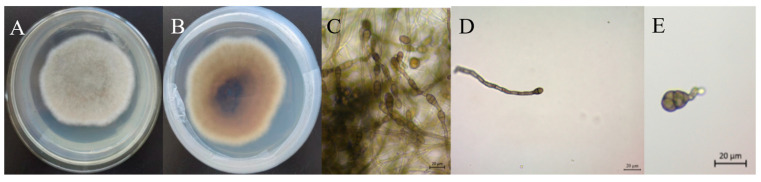
Morphological characteristics of *Alternaria* isolate SWFU-MM002 on PDA. (**A**) Colony front on PDA medium; (**B**) colony reverse on PDA medium; (**C**–**E**) spore morphology.

The isolated and purified strains were placed in the PDA medium and cultured in an incubator at 27 °C for 7d, and then we observed the colony morphology on the front side of the PDA medium (Figure 2A); at the beginning, the middle mycelium of the colony grew upward and the aerial mycelium was well developed, and with the passage of time, the colony showed a rounded shape, and the inner part of the front side of the colony was grey-brown and the outer circle was white. When observing the colony morphology on the back side of the PDA medium, it could be seen that the central part of the back side of the colony was deeper and lighter and had a whorled shape (Figure 2B). Microscopic observation showed that the spores were linked together to form a spore chain (Figure 2C). Conidia were of different morphology, mostly long ovate or inverted rod-shaped, with obvious transverse and longitudinal septa visible inside the spores, brownish-yellow in colour, and spore sizes of (11.861–26.119) μm × (6.277–12.770) μm (Figure 2D,E). Based on the morphological characteristics of colonies and conidia, strain SWFU-MM002 was initially identified as belonging to the genus *Alternaria*.

### 3.4. Phylogenetic Analysis

**Figure 3:**
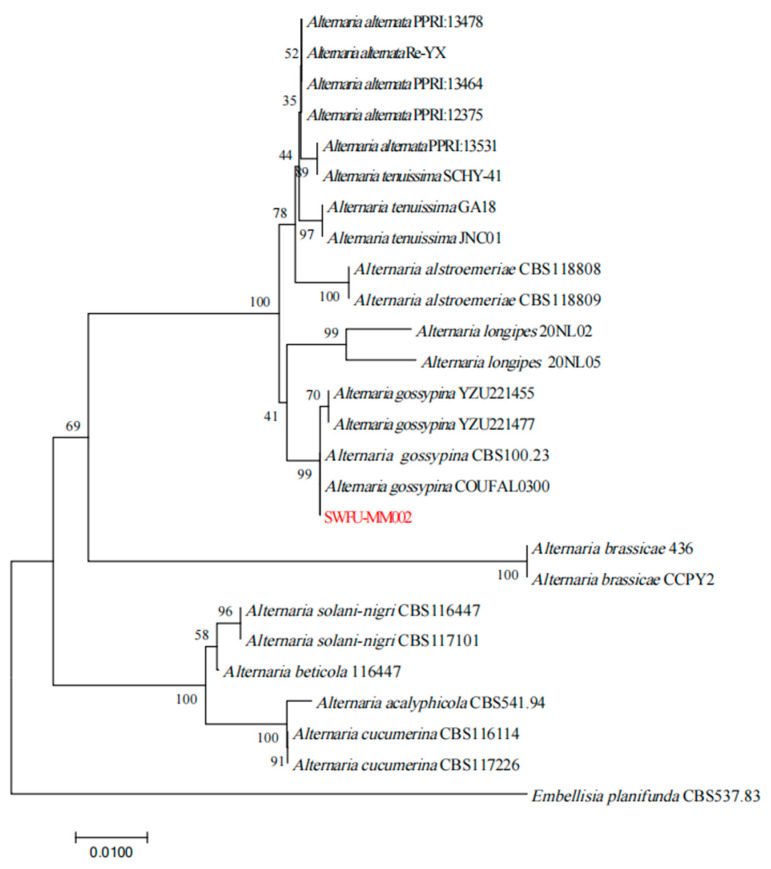
Phylogenetic tree of pathogen based on partial *ITS*, *Alta-1*, and *gpd* gene sequences. The selected isolate in this study is shown in red.

Through *ITS*, *GAPDH*, and *Alta-1* gene sequence amplification and sequencing, gene fragments with sizes of 575, 489, and 589 bp, respectively, were obtained and submitted to NCBI with entry numbers of PQ060360, PQ072896, and PQ072897. NCBI analysis showed that the homology with *Alternaria* sp. was more than 99%. By near-source comparison, this *Embellisia planifunda* can be used to construct a developmental tree. The results showed that strain SWFU-MM002 clustered in the same branch with *Alternaria gossypina* CBS 100.23 and *A. gossypina* COUFAL0300, and the support rate reached 99% (Figure 3). Combined with morphological and molecular biological characteristics, the strain was identified as *A. gossypina*.

### 3.5. Biological Characteristics

#### 3.5.1. Mycelial Growth Characteristics with Different Media

**Figure 4:**
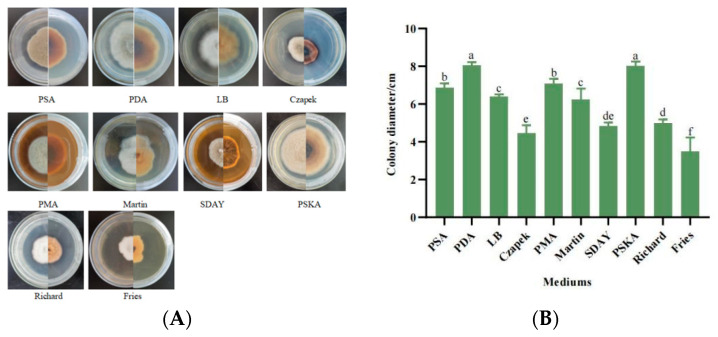
Growth of strain SWFU-MM002 under different media. (**A**) Colony growth state diagram; (**B**) culture colony diameter after 7d. Different letters indicate significant differences at *p* < 0.05 according to ANOVA.

When strain SWFU-MM002 was placed in 10 different types of media, the differences in mycelial growth were more obvious. In PDA and PSKA media, the strain grew more vigorously and the mycelium was longer, whereas in modified Fries medium, the growth was slower and the mycelium was very short. Pigment production was observed in Czapek’s medium (Figure 4A). The results showed that the most suitable media for mycelial growth were PDA medium and PSKA medium, and the colony diameters reached 8.038 cm and 8.0 cm after 7 days of incubation; Czapek culture medium, SDAY medium, and modified Fries medium were unfavourable for mycelial growth, and the colony diameters at day 7d were 4.465 cm, 4.819 cm and 3.495 cm, respectively (Figure 4B). In conclusion, different nutrients affected the mycelial growth rate during the growth of the strain and PDA medium was the most suitable medium for strain SWFU-MM002.

#### 3.5.2. Mycelial Growth Characteristics at Different pH Values

**Figure 5:**
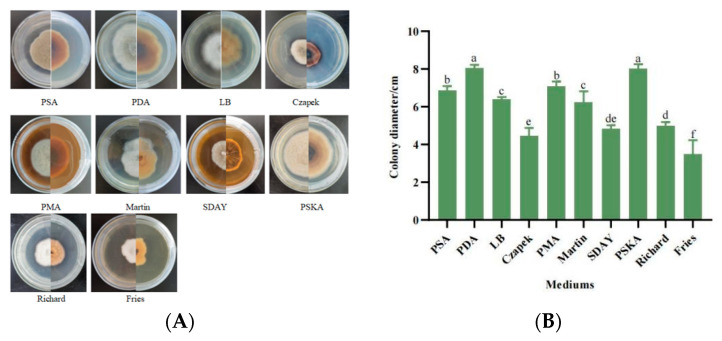
Growth of strain SWFU-MM002 under different pH values. (**A**) Colony growth state diagram; (**B**) 7d culture colony diameter. Different letters indicate significant differences at *p* < 0.05 according to ANOVA.

With the exception of the strain exhibiting slow growth at pH 4 and pH 5, there is no significant variation in growth across pH 6 to pH 9. The growth pattern appears to be relatively stable within this pH range. Notably, at pH 10, the mycelial growth is observed to be the most rapid, reaching a length of 7.05 cm by the seventh day. Conversely, under pH 4 conditions, mycelial growth is sluggish, with no visible hyphae in the initial stages of inoculation and a colony diameter of only 2.53 cm by the seventh day (Figure 5A,B). Overall, there is a positive correlation between increasing pH values and the growth rate of colonies.

#### 3.5.3. Mycelial Growth Characteristics at Different Temperatures

**Figure 6:**
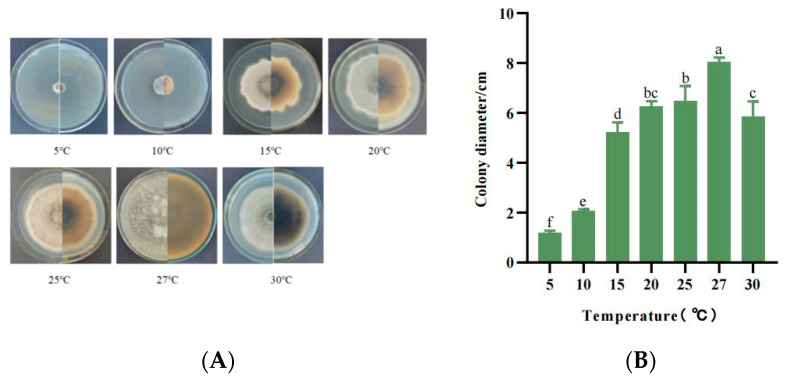
Growth of strain SWFU-MM002 under different temperatures. (**A**) Colony growth state diagram; (**B**) 7d culture colony diameter. Different letters indicate significant differences at *p* < 0.05 according to ANOVA.

The growth pattern of strain SWFU-MM002 was temperature-dependent, with growth occurring between 5 and 30 °C, but the growth rate varied greatly across the temperature range. At 5 °C, the growth of the strain was extremely slow, with the colony diameter reaching only 1.2 cm at day 7. Subsequently, mycelial growth accelerated as the temperature increased, reaching a peak at 27 °C. After 30 °C, the mycelial growth rate decreased significantly (Figure 6A,B). These results indicate that the favourite growth temperature of SWFU-MM002 was 27 °C, highlighting its sensitivity to low temperatures.

#### 3.5.4. Mycelial Growth Characteristics with Different Carbon Sources

**Figure 7:**
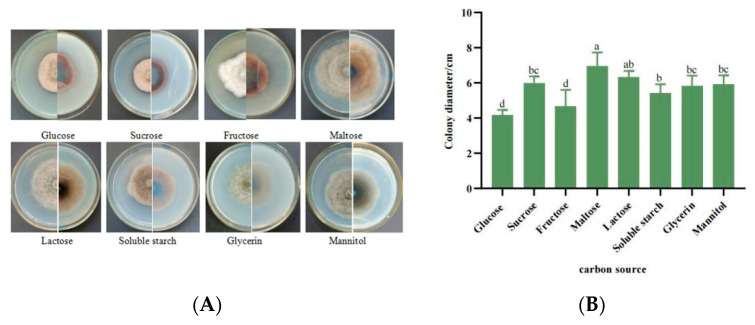
Growth of strain SWFU-MM002 under different carbon sources. (**A**) Colony growth state diagram; (**B**) 7d culture colony diameter. Different letters indicate significant differences at *p* < 0.05 according to ANOVA.

Strain SWFU-MM002 was grown in eight different carbon-source media with different growth rates and colony morphology. Most of the colonies showed rounded grey morphology, whereas colonies grown on fructose were white and irregular in shape (Figure 7A). In addition, maltose was the best carbon source for mycelial growth, with a colony diameter of 7.27 cm (Figure 7B). Among the tested carbon sources, the growth rate and colony diameter on the seventh day ranked from highest to lowest are maltose > lactose > sucrose > mannitol > glycerin > soluble starch > fructose > glucose.

#### 3.5.5. Mycelial Growth Characteristics with Different Nitrogen Sources

**Figure 8:**
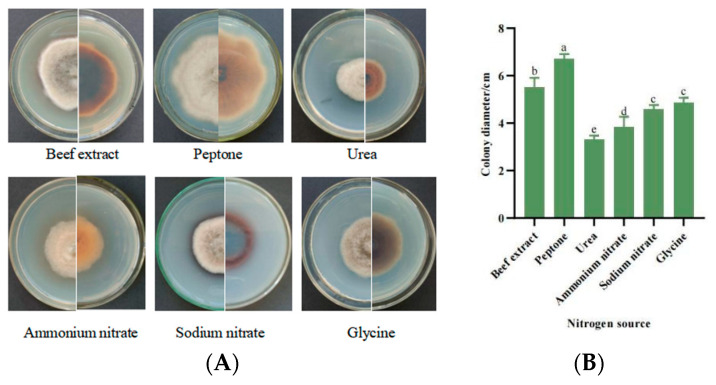
Growth of strain SWFU-MM002 under different nitrogen sources. (**A**) Colony growth state diagram; (**B**) 7d culture colony diameter. Different letters indicate significant differences at *p* < 0.05 according to ANOVA.

The growth ability of strain SWFU-MM002 was evaluated under different nitrogen-source culture conditions. The results showed that there were differences in colony morphology and size in six different nitrogen-source media (Figure 8A). The largest colony diameter of 6.72 cm was observed when peptone was used as the nitrogen source, while the slowest-growing urea colony was only 3.35 cm in diameter. Among the different nitrogen sources, mycelial growth rate and colony diameter on day 7 were ranked from largest to smallest as follows: peptone > beef extract > glycine > sodium nitrate > ammonium nitrate > urea (Figure 8B). In terms of mycelial morphology, the aerial mycelia of beef paste, peptone, sodium nitrate, and glycine were more developed than those of ammonium nitrate, which appeared to adhere to the medium. These results suggest that peptone is the most favourable nitrogen source for mycelial growth of the SWFU-MM002 strain.

#### 3.5.6. Mycelial Growth Characteristics under Different Light Conditions

**Figure 9:**
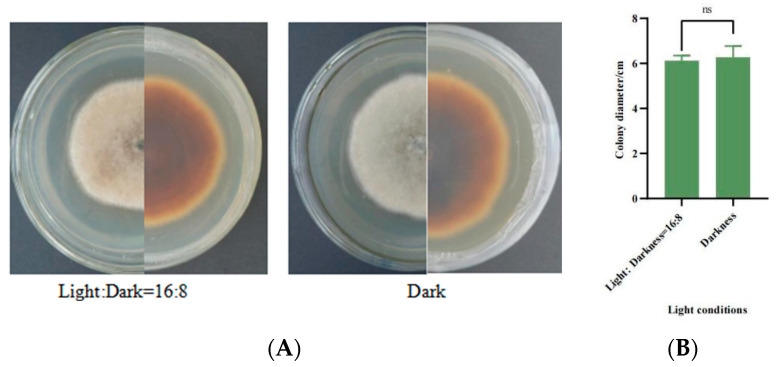
Growth of strain SWFU-MM002 under different light conditions. (**A**) Colony growth state diagram; (**B**) 7d culture colony diameter. ns = not significant.

After 7 days, the differences in the mycelial growth status of SWFU-MM002 under different light treatments were small (Figure 9A), and the differences in colony growth were not statistically significant. After 7 days of incubation, the colony diameter was 6.11 cm when the light/dark cycle was 16:8, while it was 6.27 cm under total darkness (Figure 9B). In conclusion, the light conditions had no effect on the growth of the strains.

### 3.6. Microscopic Observation of the Infection Process of Strain SWFU-MM002 in Leaves

**Figure 10:**
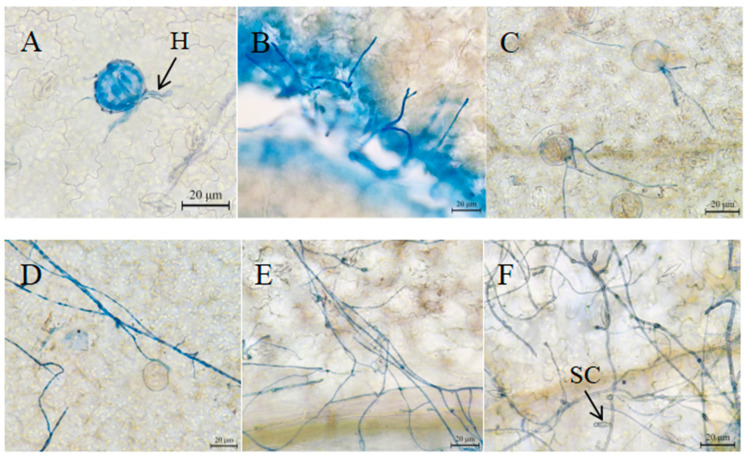
Microscopic observation of the infection process of strain SWFU-MM002. (**A**–**F**) The status of leaves 6 h, 12 h, 24 h, 48 h, 72 h and 96 h after inoculation, respectively. H: mycelia; SC: secondary conidia.

At 6 h and 12 h of inoculation, mycelia began to extend to stomata (Figure 10A,B), and at 24 h, mycelia entered the inner part of leaves from stomata (Figure 10C). With the progression of time, mycelia grew vigorously, and at 48 h and 72 h, mycelia grew in large numbers and gradually formed a network distribution (Figure 10D,E). At 96 h, the tip of some mycelia showed obvious secondary conidia (Figure 10F).

## 4. Discussion

With the continuing development of globalization and international transport, more and more invasive species are entering areas previously isolated by natural barriers [34], posing a major threat to ecosystems and ecological health, and are considered the second most important threat to biodiversity [35]. Since its arrival in China, *M. micrantha* has been increasing its area each year, reducing the yield of cash crops and seriously affecting agricultural production. It also destroys the biological communities in the invaded area [36], leading to a decrease in the population richness of the invaded area [37]. Control methods of *M. micrantha* are mainly divided into physical control and chemical control, which have controlled the spread of *M. micrantha* to some extent. However, as people’s awareness of environmental protection increases, they are more inclined to advocate for the use of biological measures to control the weed [38]. Biological control has been used and promoted because of its ability to control the disease effectively, its environmental friendliness, its lack of pollution, its lack of harm to non-target organisms, and its long-lasting effect. Currently, most biological control of *M. micrantha* uses natural enemies, parasitic insects, parasitic plants, and parasitic fungi. Among these, the parasitic fungus *Puccinia spegazzini* has received much attention. After infecting *M. micrantha*, it significantly inhibited the growth of *M. micrantha* and caused the death of *M. micrantha* when the disease was severe [14]. In the present study, *M. micrantha* was infested by the fungus *A. gossypina* and the disease index reached 75%, indicating that *A. gossypina* is highly pathogenic to *M. micrantha* leaves. In addition, it has been reported that *A. gossypina* may cause diseases in some cash crops such as *Passiflora edulis* [39], *Solanum lycopersicum* [40], tobacco [41] leaves, and other fine agricultural cash crops, but damage to forestry cash crops has not been reported. Therefore, this strain may have potential use in *M. micrantha* control.

At present, morphological observation combined with polygene sequence analysis has been widely used in the classification of *Alternaria* fungi. In this study, molecular and morphological methods were used to identify the fungi that cause leaf blight of *M. micrantha*. Based on our results, SWFU-MM002 was identified as the main pathogen of *M. micrantha* leaf blight after pathogenicity testing. The morphological and phylogenetic analysis of *ITS*, *Alta-1*, and *GAPDH* gene sequences showed that SWFU-MM002 was *A. gossypina*. The length and width of conidia are two morphological characteristics used for species differentiation [42]. The conidial sizes observed in this study match those previously described by Woudenberg et al. [43]. Combining multiple genes to identify the classification status of strains is more scientific than simple identification of a single gene sequence. Chen et al. [44] reported *Catalpa bungei* leaf spot disease caused by *A. alternata* infection through *ITS*, *TEF1*, *GAPDH*, and *RPB2* genes. Luo et al. [39] identified *A. gossypina* from stem rot of *Passilora edulis* in Guangxi, China, through *ITS*, *TEF*, *Alta-1*, *GAPDH*, *RPB2*, *SSU*, and *LSU* genes. In this study, based on the joint phylogenetic analysis of *ITS*, *Alta-1*, and *GAPDH* sequences, *A. gossypina* was identified as the pathogen of leaf spot disease of *M. micrantha*, indicating that multi-gene sequences can indeed be a good way to analyze the phylogenetic relationship of *Alternaria* fungi. Studies have shown that *Alternaria* fungi can cause a variety of weed diseases, such as *Xanthium*, *Convolvulus chinensis* [31], *Rumex crispus* [45], *Echinochloa crusgalli* [46], *Datura stramonium* [47], and *Ageratina adenophora* [48]. This study identified *A. gossypina* as the pathogen causing the leaf disease of *M. micrantha*. This was the first report of brown spot caused by *A. gossypina*’s infection with *M. micrantha*.

An important feature of fungal herbicides is that they have strict requirements on environmental conditions, and only under sufficient moisture and appropriate temperature conditions can they ensure effective infection and good control [49]. It has been reported that *A. gossypina* has good colony growth on PDA and OA media, and can grow on a variety of fungal media, indicating that *A. gossypina* has relatively low nutritional requirements [50]. In this study, the strain SWFU-MM002 could grow on PDA, PSA, PSKA, PMA, and other media; its growth on PDA is the best and growth on maltose and peptone as carbon and nitrogen sources is better, indicating that the strain is suitable for large-scale culture, which provides a basis for low-cost large-scale artificial culture. In addition, *Alternaria* has a wide adaptability to temperature, which plays an important role in sporulation, spore germination, and subsequent infection of *Alternaria*. The optimum growth temperature of *A. tenuissima* was 28 °C [42], and the optimum growth temperature of *A. alternata* was 25 °C [51]. The optimum growth temperature of *A. solani* is 27 °C [52], and the optimum growth temperature of *A. brassicae* is 15–20 °C [53]. The optimum growth temperature of strain SWFU-MM002 in this study is 27 °C. When the PH was between 7 and 10, mycelia grew strongly, indicating that the SWFU-MM002 strain was more suitable for growth under alkaline conditions. In addition, SWFU-MM002 was not sensitive to light conditions, and there was no significant difference in mycelium growth under different light conditions. In summary, this strain is very conducive to the application of biological control in terms of biological characteristics and is a plant pathogenic fungus with a very promising application.

The study of the fungal infection process is helpful for us to understand the process of fungal diseases. Studies have shown that stomata are the main route for many plant pathogens to enter plant tissues [54]. In this study, it was observed that some mycelia entered the tissue through stomata 24 h after infection. After 72 h, mycelia extended longitudinally and transversely on the leaf surface and branched freely to form a network structure. By observing the development of disease symptoms on leaves during infection, it is speculated that mycelium invades leaf tissues after cell death and obtains nutrients from dead or dying cells to further spread and grow [55]. The strains may destroy the cell structure of the host plant by increasing intracellular pressure or producing toxins to destroy the cell structure, thus completing the infection process.

Obtaining biocontrol strains with potential for bioherbicide development is the premise and basis of bioherbicide development, but there is still a lot of work to be carried out before the successful development of a commercial bioherbicide. For example, the optimization of fermentation conditions of the SWFU-MM002 strain and how to further improve the spore production and pathogenic ability of SWFU-MM002 need to be further studied. In addition, the herbicidal active substances in the metabolites of SWFU-MM002 can be extracted and identified, and the biosynthetic pathway of the herbicidal active ingredients and the functions of key metabolic genes can be clarified, providing a theoretical basis and scientific reference for the development of new biological herbicide targets.

## 5. Conclusions

In summary, through morphological observation, molecular biological identification, and pathogenicity study, the pathogenic fungus causing a large number of necrotic injuries to *M. micrantha* leaves was identified as *A. gossypina*. This is the first report on leaf spot of *M. micrantha* caused by *A. gossypina*. The optimal medium for mycelia growth of the strain SWFU-MM002 was PDA medium and PSKA medium; the optimal temperature was 27 °C; and the optimal pH was 6–10. The optimal carbon source and nitrogen source were maltose and peptone, respectively. This strain has an inhibitory effect on the growth of the invasive species *M. micrantha*. The research results clarified the culture characteristics of this strain, provided a theoretical basis for the subsequent development of this strain as a biological control agent for *M. micrantha*, and provided a new research direction for the new green control of *M. micrantha*.

## Figures and Tables

**Table 1:** Disease intensity assessment scale in this study.

Class	Rank/Numerical Value	% of Infected Leaf Area
I	0	Disease free
II	1	1–10
III	2	11–25
IV	3	26–50
V	4	51–75
VI	5	>76

**Table 2:** Primers for PCR used in this study.

Gene/Sequence	Primer	Primer Sequence (5′→3′)
*ITS*	ITS1	TCCGTAGGTGAACCTGCGG
	ITS4	TCCTCCGCTTATTGATATGC
*gpd*	gpd1	CAACGGCTTCGGTCGCATTG
	gpd2	GCCAAGGAGTTGGTTGTGC
*Alta-1*	Alt-for	ATGCAGTTCACCACCATCGC
	Alt-rev	ACGAGGGTGAYGTAGGCGTC

## Data Availability

Data are contained within the article and Supplementary Materials.

## Supplementary Materials

The following supporting information can be downloaded at https://www.mdpi.com/article/10.3390/jof10100691/s1: Table S1: Strains and multi-gene GenBank accession numbers for phylogenetic analysis.
