# Correction: Feng et al. Identification and Biological Characteristics of *Alternaria gossypina* as a Promising Biocontrol Agent for the Control of *Mikania micrantha*. *J. Fungi* 2024, *10*, 691

**DOI:** 10.3390/jof11120872

**Published:** 2025-12-09

**Authors:** Lichen Feng, Lianrong Hu, Jingyi Bo, Mei Ji, Sangzi Ze, Yan’e Ding, Bin Yang, Ning Zhao

**Affiliations:** 1 College of Biological Science and Food Engineering, Southwest Forestry University, Kunming 650224, China; flc225169@163.com (L.F.); 15223788948@163.com (J.B.); longnanding@163.com (Y.D.); 2 Yunnan Academy of Forestry and Grassland, Kunming 650224, China; hulianrong@yafg.ac.cn (L.H.); meiji.emma@163.com (M.J.); 3 Yunnan Forestry and Grassland Pest Control and Quarantine Bureau, Kunming 650051, China; zesangzi@163.com; 4 School of Biological and Chemical Science, Pu’er University, Pu’er 665000, China; 5 Key Laboratory of Forest Disaster Warning and Control of Yunnan Province, Southwest Forestry University, Kunming 650224, China

**Error in Figure**

In the original publication [1], due to an error made by the editorial office, there was a mistake in Figure 5 as published; the author’s final proofread version differs from Figure 5 in the published paper. Figure 5 should depict the growth of strain SWFU-MM002 under different pH values (including Figure 5A: Colony growth state diagram; Figure 5B: 7d culture colony diameter), rather than its growth on different media. The specific reason is that during the manuscript production stage, Figure 5 in the image package was incorrect, resulting in the final version of Figure 5 being incorrect. The corrected Figure 5 is shown below. The authors state that the scientific conclusions are unaffected. The original publication has also been updated.

## Figures and Tables

**Figure 5:**
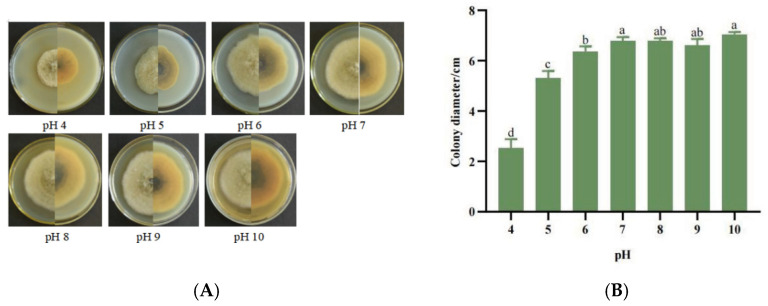
Growth of strain SWFU-MM002 under different pH values. (**A**) Colony growth state diagram; (**B**) 7d culture colony diameter. Different letters indicate significant differences at *p* < 0.05 according to ANOVA.
