# Supplementary material for: Identification and Biological Characteristics of Alternaria gossypina as a Promising Biocontrol Agent for the Control of Mikania micrantha
Source: J Fungi (Basel). 2024 Oct 3;10(10):691. doi: 10.3390/jof10100691 (PMC11508739; doi:10.3390/jof10100691)
Supplement: Supplementary file 1 [file jof-10-00691-s001.zip › jof-3222542-supplementary.pdf]

Table S1. Strains and multi-gene GenBank accession numbers for phylogenertic analysis

Species	StrainNo.	GenBank accessionNo.		
		ITS	Alta1	gpd
Alternaria alternata	PPRI:13531	MF381815	MF381763	MF381789
A. alternata	PPRI:13478	MF381802	MF381750	MF381776
A. alternata	PPRI:13464	MF381796	MF381744	MF381770
A. alternata	PPRI:12375	KY099685	KY099647	KY099666
A. alternata	Re-YX	OP559163	OP575313	OP575315
A. gossypina	YZU 221455	OR435098	OR455818	OR270927
A. gossypina	YZU 221477	OR710806	PP057865	PP057862
A. gossypina	COUFAL0300	MT522406	MT530380	MT530366
A. gossypina	CBS 100.23	KP124429	KP123977	KP124280
A. acalyphicola	CBS 541.94	KJ718097	KJ718617	KJ717952
A. alstroemeriae	CBS 118808	KP124296	KP123845	KP124153
A. alstroemeriae	CBS 118809	NR_163686	MH084526	KP124154
A. beticola	116447		JQ905105	JQ905162
A. tenuissima	SCHY-41	ON318277	ON352172	ON352292
A. tenuissima	JNC01	PP350760	PP746506	PP409573
A. tenuissima	GA18	MZ314834	MZ330656	MK451974
A. longipes	20NL02	OK426388	OK469304	OK469302
A. longipes	20NL05	OK426389	OK469305	OK469303
A. brassicae	436	KP993531	KR051382	KR051390
A. brassicae	CCPY2	MG250600	MG250636	MG250612
A. cucumerina	CBS 116114	KJ718153	KJ718668	KJ718000
A. cucumerina	CBS 117226	KJ718155	KJ718670	KJ718002
A. solani-nigri	CBS 116447	KJ718246	KJ718752	KJ718074
A. solani-nigri	CBS 117101	KJ718247	KJ718753	KJ718075
Embellisia planifunda	CBS 537.83	FJ357315	FJ266507	FJ357303
